# Evidence of non-transferrin-bound iron in patients with ST-elevation myocardial infarction: relationship with microvascular obstruction and post-reperfusion myocardial hemorrhage

**DOI:** 10.1186/1532-429X-17-S1-P118

**Published:** 2015-02-03

**Authors:** Alberto Roghi, Erika Poggiali, Lorena Duca, Antonio Mafrici, Patrizia Pedrotti, Stefania Paccagnini, Sergio Brenna, Alessio Galli, Dario Consonni, Maria Domenica Cappellini

**Affiliations:** Cardiology and Cardiovascular Surgery A.De Gasperis, Niguarda Ca’Granda Hospital, Milan, Italy; Clinical Sciences and Community Health, University of Milan, Milan, Italy; Medicine and Medical Specialties Ca’Granda Foundation, Ospedale Maggiore, Milan, Italy; Cardiology Unit, San Carlo Borromeo Hospital, Milan, Italy; Biochemestry Laboratories, Niguarda Ca’Granda Hospital, Milan, Italy; Cardiovascular Disease Unit, Ca’Granda Foundation IRCCS, Ospedale Maggiore, Milan, Italy; Rare Disease Center, Department of Medicine and Medical Specialties, University of Milan, Milan, Italy

## Background

Hereditary hemochromatosis, thalassemia and myelodysplastic sindromes represent iron toxicity models with evidence of iron-related heart failure. Non-Transferrin-Bound Iron (NTBI), plays a key role in the pathogenesis of cardiac toxicity leading to the production of reactive oxygen species which increase lipid peroxidation (MDA). In acute myocardial infarction (MI) microvascular obstruction (MVO) and hemorrhage (HEM) are independent predictors of left ventricular (LV) remodelling. HEM may be a source of iron toxicity through NTBI and pro-inflammatory mediators (IL-6) , which can directly contribute to acute impairment of myocardial function and adverse LV remodelling.

The aim of the study was to assess NTBI in a consecutive cohort of patients (pts) prospectively enrolled with ST-elevation MI (STEMI).and its relationship with MVO and HEM.

## Methods

Fifteen pts with STEMI were enrolled if the onset of symptoms had been < 12 hours before PCI. NTBI, MDA and IL-6 were assessed at admission and at 0-3-6-9-12-24 hours. Cardiac Magnetic Resonance imaging (CMR) was performed on a 1.5 T scanner at 5 days and 6 months after the cardiac event. Myocardial edema and HEM were assessed by T2 and T2* mapping. MVO, myocardial salvage and necrotic area were assessed by early and late gadolinium enhancement (LGE).

## Results

NTBI was detected in 13/15 pts with highest values in 4 pts with evidence of MVO and HEM. NTBI levels were significantly related to CK-MB and troponin T values. NTBI kinetic appears different in MVO and HEM (7/15 pts), with a peak values at 6 hours after PCI, in comparison with those with no evidence of MVO and HEM, in whom NTBI values were lower and remained indeterminable after the first 24 hours.

## Conclusions

The detection of elevated NTBI values in pts with STEMI, MVO and HEM suggest a possible role of iron cardiotoxicity in myocardial damage.

## Funding

None.

